# Demographics and clinical characteristics of carbon monoxide poisoning for patients attending in the emergency department at a tertiary hospital in Riyadh, Saudi Arabia

**DOI:** 10.1186/s12245-024-00600-w

**Published:** 2024-02-26

**Authors:** Nesrin Alharthy, Aljohara Alanazi, Alreem Almoqaytib, Bedour Alharbi, Rakad Alshaibani, Jawaher Albuniyan, Abdullah Alshibani

**Affiliations:** 1 Pediatrics Emergency Department, King Abdulaziz Medical City, Riyadh, Saudi Arabia; 2 Emergency Medical Services Department, College of Applied Medical Sciences, King Saud bin Abdulaziz University for Health Sciences, Riyadh, Saudi Arabia; 3 King Abdullah International Medical Research Center, Riyadh, Saudi Arabia

**Keywords:** Intoxication, Risk factors, Delayed neuropsychiatric sequelae, Emergency care, Descriptive analysis

## Abstract

**Background:**

Carbon Monoxide (CO) is one of the most common environmental causes of acute intoxication globally. It can lead to the development of Delayed Neuropsychiatric Sequelae (DNS) which may develop in 2-40 days after remission of acute CO poisoning. DNS is defined by recurrent-transient neurological, cognitive, or psychological manifestations. This study was intended to describe the demographics and characteristics of CO poisoning patients attending at the Emergency Department (ED) and assess the association between CO intoxication and the development of DNS in a tertiary hospital, Riyadh, Saudi Arabia.

**Methods:**

A retrospective descriptive cross-sectional study was conducted in subjects who were diagnosed with CO poisoning and attended to the ED at King Abdulaziz Medical City (KAMC) and King Abdullah Specialist Children’s Hospital (KASCH) in Riyadh during the period from January 2016 to December 2021. Patient demographics, vitals, diagnostic tests, and oxygen therapy at initial presentation were documented. Patient medical records were reviewed at 2-40 days following CO poisoning for development of DNS. Ethical approval was obtained from King Abdullah International Medical Research Center (KAIMRC).

**Results:**

A total of 85 patients were diagnosed with CO poisoning and met the study inclusion criteria. Of those, 76% were adults with an average age of 32.36 (SD ± 15.20) and 51% were male adults. Five (6%) of the 85 patients developed DNS. Common symptoms included dizziness, nausea, and decreased visual acuity in 40% of the cases. The development of DNS manifestations was most likely (80%) to occur at 2 to 10 days after the initial incident. Inferential statistics showed that BMI (*p*-value = 0.021) and age group (p-value = 0.029) were significantly associated with COHb level, which was not the case for gender and the presence of clinical manifestation. Furthermore, Gender was significantly associated with the development of DNS (20% male vs. 80% female, *p* = 0.050).

**Conclusions:**

The findings of this study are consistent with previous published studies showing low proportions of patients who were exposed to CO poisoning at risk of developing DNS. Further larger-scale multicenter studies are needed to assess the factors associated with the development of DNS for patients with CO poisoning.

## Introduction

Carbon monoxide (CO) poisoning is a true emergency that is associated with high mortality rates. CO poisoning has a global annual incidence and mortality rate of 137 cases per million people and 4.6 deaths per million people, respectively [1]. Over the last 25 years, the global annual incidence rate of CO poisoning has remained relatively constant, while the risk of death decreased between 1992 and 2017 by 36% (odds ratio, 0.64; 95% confidence interval, 0.44–0.92; *p*-value =0.018) from 7.2 cases to 4.6 cases per million people [1]. With regards to the Middle East and North Africa, a recent systemic review found that there was a total of 19,726 diagnosed CO poisoning cases, of which, 1722 cases resulted in death (9.6%) [2].

CO poisoning is diagnosed clinically, and requires a history of recent CO exposure, development of symptoms associated with CO poisoning, and the presence of an elevated carboxyhemoglobin level (COHb) [3]. Increased COHb level can be used to confirm the clinical diagnosis of acute CO poisoning [3]. According to the World Health Organization (WHO), levels of more than 6 ppm are potentially harmful over a prolonged period of time [4]. COHb levels higher or equal to 2% or higher in nonsmokers and 10% or higher in smokers are considered above the normal level and may cause symptoms [4].

CO poisoning is associated with serious complications including, but not limited to, neurological complications, such as Delayed Neuropsychiatric Sequelae (DNS) [5]. DNS is described as recurrent-transient neurological, cognitive, or psychological complications following CO intoxication [6]. Up to 40% CO poisoning cases, DNS occurs within a few weeks of initial remission (within 6 weeks, greatest risk at week-period) of acute poisoning [7]. Long-term exposure to CO and CO toxicity from burning charcoal were identified in recent studies as risk factors of developing DNS [6, 8].

Literature exploring and assessing the incidence rates and associated outcomes with CO poisoning and the development of DNS in Saudi Arabia is limited. Therefore, this study aims to assess the demographics and characteristics of patients with CO poisoning. It will also explore and assess the development of DNS in patients who survived after initial exposure to CO poisoning and the potential risk factors.

## Methods

### Study design and setting

This study is a retrospective cross-sectional chart review of all patients diagnosed with CO poisoning and admitted to King Abdulaziz Medical City (KAMC) and King Abdullah Specialist Children’s Hospital (KASCH) in Riyadh city, Saudi Arabia from 01st January 2016 to 31st December 2021. KAMC is Riyadh city is considered as one of the most comprehensive healthcare facilities in Saudi Arabia. It provides all levels of healthcare from primary care and public health to advanced tertiary services. At present, KAMC has 1973 operational beds and over 11,000 healthcare workers. KASCH is an advanced pediatric tertiary care center and one of the pediatric specialist hospitals in Saudi Arabia. It provides all ranges of healthcare to pediatric patients from outpatient clinics to advanced tertiary inpatient diagnostics and therapeutics. KASCH currently has around 550 beds in total for inpatients and 60 beds for ED and trauma.

### Study participants

The study included both adult and pediatric patients who were presented at the ED of both KAMC and KASCH with CO poisoning in the time period from 1st January 2016 to 31st December 2021. Patients who were known to have neuropsychiatric manifestations before CO poisoning were excluded from the study. Patients transferred from another hospital were also excluded. Thus, all patients meeting the inclusion criteria were included in the study.

### Data collection

Data were retrieved from the “Best Care System”, Which is an electronic medical record system that is used for all patients attending at KAMC and KASCH. All data were collected and documented using a predetermined data collection form. Data items included baseline variables including age, gender, Body Mass Index (BMI), smoking status, weather condition, and type of exposure. BMI stratification was based on the literature where a score under 18.5 is underweight, a score of 18.5-24.9 is normal, overweight 25-29.9 and over 30 score is obese [9, 10]. Smoking status was assessed based on the patient time of presentation. Therefore, patients who reported smoking are the ones who are currently smoking. For the weather condition, cold weather in Saudi Arabia starts from December until March, and hot weather starts from April to November. In addition to baseline data, the presence of clinical manifestations including the signs and symptoms related to Central Nervous System (CNS), respiratory system, and cardiovascular system were collected. Moreover, Arterial Blood Gas (ABG) Findings and COHb level were collected and reported in this study. For the COHb level, published reports showed that normal carboxyhemoglobin level is low as 2% for non-smokers and 5% for smokers [9]. In this study, we selected the carboxyhemoglobin cutoff level to be 5%. Less than 5% considered to be normal, and more than 5% abnormal [9]. The files of CO poisoning patients were reviewed for up to 40 days following their presentation at the ED for any development of DNS based on the literature reporting peak period of 6 weeks [11–13]. The onset and type of DNS developed were also documented. Data was collected by the study team. To ensure accuracy, the supervisor of the study reviewed the collected data by the study team. The collected data were stored and kept secured at a university desktop of the study supervisor in a password-protected file where only the study team has access to this data. The study obtained institutional review board approval from King Abdullah International Medical Research Center (KAIMRC).

### Data analysis

The collected data were entered into a Microsoft Excel sheet and, then, moved to SPSS 20 for analysis. Shapiro-Wilk test was used for normality. Categorical variables including age, gender, BMI, smoking status, weather condition, type of exposure, and presence of clinical manifestations including the presence of the CNS, respiratory system, and cardiovascular system manifestations were presented using proportions and percentages. ABG findings and COHb level of the study population were presented using Median (Inter-quartile Range [IQR]). Baseline characteristics and clinical information were assessed and presented for all study population and then stratified by age groups to adult and pediatric patients. Age in years and BMI in kg/m2, as they were not normally distributed, were presented in Median (IQR) to assess their association with the development of DNS. The study reported the DNS as categorical due to limited information on time to event. The Mann Whitney U test was used to assess the association between the COHb level for non-parametric variables. Pearson Chi square test Fisher’s Exact test was used to assess the association between the COHb level as a categorical variable with baseline variables. The association between baseline variables and the development of DNS was assessed using Mann Whitney U test, Pearson Chi square test, and Fisher Exact test. A *p*-value of < 0.05 was predetermined to be considered statistically significant in this study.

## Results

### Characteristics of the study population

Based on the total sample size, 85 patients were diagnosed with CO poisoning and met the study inclusion criteria. Of these, 61 (71.8%) were adults with an average age of 32.36 (SD ± 15.20) and 55 (64.7%) were males (Table 1). Thirty (35.29%) of the study population had normal BMI (Table 1). None of pediatric patients were reported to be smokers (Table 1). Of adult patients, 21 (34.4%) were reported to be smokers while the majority (65.6%) of adult patients had unknown smoking status (Table 1).

**Table 1:** Demographic details of study population

Variables	Total (***n*** = 85)	Pediatrics (***n*** = 24)	Adults (***n*** = 61)
	Frequency (Percentage)		
**Gender**			
Males	55(64.7)	14(58.3)	41(67.2)
Females	30(35.3)	10 (41.7)	20(32.8)
**Weather condition**			
Cold weather	65(76.5)	22(91.7)	43(70.5)
Hot weather	20(23.5)	2(8.3)	18(29.5)
**Type of exposure**			
Open Space	4(4.71)	0	4(6.6)
Enclosed Space	71(83.53)	23(95.8)	48(78.7)
Unknown	10(11.76)	1(4.2)	9(14.8)
**Body Mass Index**			
Underweight	11(12.94)	11(45.8)	0
Normal	30(35.29)	6(25)	24(39.3)
Overweight	12(14.12)	2(8.3)	10(16.4)
Obese	6(7.06)	1(4.2)	5(8.2)
Not Applicable	26(30.59)	4(16.7)	22(36.1)
**Smoking status**			
Yes	21(24.7)	0	21(34.4)
No	24(28.2)	24(100)	0
Unknown	40(47.1)	0	40(65.6)
**Clinical Manifestations**			
Presented with clinical manifestations	78(91.8)	17(70.8)	61(100)
Presented with No clinical manifestations	7(8.2)	7(29.2)	0
**CNS symptoms**			
Presented with CNS symptoms	74(87.1)	16(66.7)	58(95.1)
Presented without CNS symptoms	11(12.9)	8(33.3)	3(4.9)
**Respiratory symptoms**			
Presented with Respiratory symptoms	44(51.8)	7(29.2)	37(60.7)
Presented without Respiratory symptoms	41(48.2)	17(70.8)	24(39.3)
**Cardiovascular symptoms**			
Presented with cardiovascular symptoms	28(32.9)	4(16.7)	24(39.3)
Presented without cardiovascular symptoms	57(67.1)	20(83.3)	37(60.7)

**Fig. 1:**
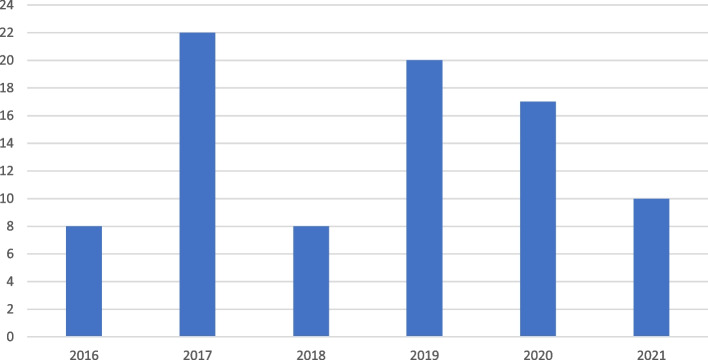
Number of carbon monoxide poisoning cases over the study years

Most (76%) of CO incidents were encountered during the winter period from November to January (peaked in December). More information about the number of encountered CO cases based on months is available in Fig. 1. Furthermore, such incidents mostly (83.5%) occurred in enclosed spaces for all study population (Table 1). Enclosed spaces stayed the most common type of exposure to CO poisoning when the study population was stratified by age group (adults [78.8%] and pediatrics [95.8%]) (Table 1).

### Information about vital signs

The main ABG findings (pH, PO2, PCO2, and HCO3) and COHb level of the study population at their initial presentation in the hospital are presented in Table 2. Median (IQR) for each variable was presented for the whole study population and then stratified to adult and pediatric patients (Table 2). The median COHb level of the study population was 12.8 (3.7, 20.1) and was higher in pediatric patients compared to adult patients (Table 2). Adult patients, when compared to pediatric patients, had higher PO2 level (33.9 [21.9, 53.4] vs. 29.9 [26.10, 49.3]) (Table 2). Both adult and pediatric patients showed almost similar PCOs, pH, and HCO3 findings (Table 2). Oxygen therapy at the ED was mostly performed through the application of non-rebreather mask (59.5% of all cases), followed by intubation and mechanical ventilation (2.6%), simple face mask (2.6%), and nasal cannula (0.9%). There were eight cases (6.8%) where no oxygen therapy was reported.

**Table 2:** Information on vital signs for the study subjects

Variable	Median and IQR		
	Adult	Pediatric	Total
COHb	10.7(3.65, 19.8)	14.1(6.18, 21.88)	12.8(3.7, 20.1)
PO2	33.9(21.9, 53.4)	29.9(26.10, 49.3)	32.96(22.85, 52.57)
PCO2	43.7(37.7, 47.6)	43(39.6, 47.3)	43.35(39.08, 47.38)
Ph	7.37(7.33, 7.39)	7.36(7.33, 7.39)	7.36(7.33,7.39)
Bicarbonate	24.4(22, 26.4)	23.6(22.2, 25.4)	24(22.1, 25.97)

### Presence of clinical manifestations

The majority (91.8%) of the patients showed clinical manifestations of CO poisoning, of which all adult patients (100%) presented with manifestations. Most patients were more likely to show symptoms related to the central nervous system (87.1%) followed by respiratory system symptoms (51.8%) cardiovascular system symptoms (32.9%). This stayed the same when the study population was stratified by age groups.

A broad range of signs and symptoms related to central nervous, respiratory, and cardiovascular systems were reported for patients with CO poisoning (Table 3). For patients with central nervous system symptoms, the most common reported symptom was headache (67.6% of the cases) followed by dizziness (54.1% of the cases) (Table 3). Only 1 (1.4%) patient presented with generalized weakness (Table 3). Dyspnea was, by far, the most common reported symptom for patients with respiratory system symptoms (86.4% of the cases) followed by coughing and tachypnea (13.6% of the cases for each symptom) (Table 3). For patients with cardiovascular system manifestations, 71.4% of the cases reported chest pain symptoms followed by hypertension (17.9% of the cases) (Table 3).

**Table 3:** Proportions of CO poisoning patients based on clinical manifestations

Clinical manifestations	Frequency^**a**^(Percentage)
**1. Central Nervous System** Headache Dizziness Nausea Abnormal movement Confusion Vomiting Altered level of consciousness Numbness Seizure Comma Weakness	**74(87.1)** 50(67.6) 40(54.1) 19(25.7) 1(1.4) 9(12.2) 17(23) 29(39.2) 3(4.1) 7(9.5) 1(1.4) 1(1.4)
**2. Respiratory System** Dyspnea Cough Hypoxia Tachypnea Voice Change	**44(51.8)** 38(86.4) 6(13.6) 1(2.3) 6(13.6) 1(2.3)
**3. Cardiovascular System** Chest pain Palpitation Diaphoretic Hypertension Fainting Cardiac Arrest	**28(32.9)** 20(71.4) 2(7.1) 3(10.7) 5(17.9) 1(3.6) 1(3.6)

^a^The study subjects can have more than one manifestation

### Development of delayed neuropsychiatric sequela (DNS)

Of patients who were initially exposed to CO poisoning, only five out of 85 patients (6%) developed DNS. The reported symptoms for patients with DNS included dizziness, nausea, and decreased visual acuity in 40% (n = 2/5) of the cases. Other manifestations were weakness, vertigo, palpitation, numbness, vomiting, and brain death which were reported in 20% (*n* = 1/5) of the cases. One (20%) of the five cases developed DNS manifestations in less than 2 days, while the remaining four cases (80%) (*n* = 4/5) developed the symptoms between 2 to 10 days after the initial incident. Three out of the five DNS cases received oxygen therapy at the ED through non-rebreather mask, one through intubation and mechanical ventilation, and one had no reported oxygen therapy. Of the total cases (*n* = 85) included in this study, only one death was reported. This case developed a DNS within 2-40 days after exposure to carbon monoxide poisoning and death occurred within this period.

### Association between COHb level and baseline variables

The study assessed the association between COHb level with baseline variables of patients with CO poisoning. These variables included BMI, gender, age group, and presence of clinical manifestations (Table 4). When the COHb level was handled as a continuous variable, none of the baseline variables was significantly associated with the COHb level except for gender (Male median [IQR], 15.75 [7.42, 22.57]) versus (Female median [IQR],10.6 [2.2, 15.4]) (*p*-value =0.004). When the COHb level was handled as a categorical variable, BMI (p-value = 0.021) and age group (p-value = 0.029) were significantly associated with COHb level (Table 5). Other variables were not statistically significant (Table 5).

**Table 4:** The association between Carboxyhemoglobin level (continuous variable) and other variables (%) in patients with CO poisoning; (*n* = 85)

Variable	Carboxy Hb level(%) Median (IQR)	P-value
**BMI (in kg/m2)**		
Underweight Normal Overweight Obese	16(2.7, 22.7) 10.7(5.4, 18.7) 9.7(4.5, 21.6) 7.25(1.4, 20.97)	0.687
**Gender**		
Males Females	15.75(7.42, 22.57) 10.6(2.2, 15.4)	0.004*
**Age group**		
Pediatric Adult	14.4(6.17, 21.87) 10.7(3.65, 19.8)	0.609
**Clinical Manifestations**		
Yes No	11.4(3.65, 19.95) 14.55(7.35, 22.87)	0.667

Mann Whitney U test was used, *Statistically Significant at 5%

**Table 5:** The association between Carboxyhemoglobin level (categorical variable) and other variables (%) in patients with CO poisoning; (*n* = 85)

Variable	Carboxy Hemoglobin			***P*** value
	Normal	Abnormal	Total	
**BMI (in kg/m2)**^**b**^				
Underweight	0	11(100)	11(100)	
Normal	6(24)	19(76)	25(100)	
Overweight	3(25)	9(75)	12(100)	0.021*
Obese	4(66.7)	2(33)	6(100)	
**Gender**^**a**^				
Males	12(25)	36(75)	48(100)	0.301
Females	4(14.8)	23(85.2)	27(100)	
**Age group**^**b**^				
Pediatric	1(4.5)	21(95.5)	22(100)	0.029*
Adult	15(28.3)	38(71.7)	53(100)	
**Clinical Manifestations**^**b**^				
Yes	16(23.2)	53(76.8)	69(100)	
No	0	6(100)	6(100)	0.331
**Exposure Type**				
Open Space	2(50)	2(50)	4(100)	
Closed Space	12(19.4)	50(80.6)	62(100)	0.351
Unknown	2(22.2)	7(77.8)	9(100)	

^a^Pearson Chi square test, ^b^Fisher Exact test, *Statistically Significant at 5%

### Association between baseline variables and the development of DNS

The association between age, BMI, type age, gender, type of exposure, presence of clinical manifestations, and COHb level and the development of DNS was assessed using appropriate statistical analyses (Table 6). The findings showed no statistically significant association between any of these variables with the development of DNS. However, gender was clinically as one male (20%) developed DNS compared to four females (80%), showing statistically a p-value of 0.050 (Table 6). In this study, patients who developed DNS within 40 days after presentation to ED were reported. No information if some or all patients developed permanent sequaele.

**Table 6:** The association between age, gender, COHb level, and Exposure type with the development of DNS; (*n* = 85)

Variables	With Delayed Neuropsychiatric sequelae, n (%)	No Delayed Neuropsychiatric sequels, n (%)	***P***-value
**Gender (including adult and pediatric)**			
Male	1(20)	54(67.5)	0.050
Female	4(80)	26(32.5)	
**Age in years**, Median (IQR)	24(6.5, 28)	26(16, 35.25)	0.287
BMI in kg/m2, Median (IQR)	20.51(17.71, 27.66)	24.22(17.07, 27.41)	0.809
**Type of exposure**			
Open Space	1(20)	3(3.8)	0.366
Enclosed space	4(80)	67(83.8)	
Unknown	0(0)	8(10)	
Others	0(0)	2(2.5)	
**Presence of Clinical Manifestations**			
Yes	5(100)	73(91.2)	1.000
No	0	7(8.8)	
Carboxy hemoglobin level	6.35(1.32, 16.32)	13(4.9, 20.3)	0.179

Mann Whitney U test, Pearson Chi square test and Fisher Exact test were used

## Discussion

This study has assessed the characteristics and patterns of CO poisoning and the development of DNS. Adults (71.8%) and males (64.7%) were more commonly to present with CO poisoning. Most cases were encountered during winter and in enclosed spaces. Almost 92% of the study population presented with clinical manifestations. CNS related manifestations were the most common signs/symptoms of CO poisoning. Of the whole study population, five (6%) patients developed DNS. COHb level was significantly associated with gender, BMI, and age group. There was no significant statistical association between the development of DNS and demographics and baseline information, except for gender which showed clinically significant association with the development of DNS (20% male vs. 80% female, *p* = 0.050).

### Evidence from national literature

Although limited, the available evidence from Saudi Arabia with regards to CO poisoning and the development of DNS is mostly consistent with the findings of this study. Findings from the national studies showed that CO poisoning was more commonly prevalent in males (male: female ratio of 3.8:1) [5] and most deaths resulting this condition occurred for males (82%) [14, 15]. The mean age of patients presenting with CO poisoning was 35.5 years (+ 12.5) [5], close to our findings (mean age was 32.36 [± 15.20]). Adults were more likely to be present with CO poisoning than pediatrics, consistent with our findings [14].Findings from national studies were also consistent with our findings, showing that most CO poisoning cases occurred during winter period (ranging between 50 and 90%) and at home (ranging between 79 to 88%) [14, 15].

Clinical manifestations resulting from CO poisoning were commonly related to CNS system [5], consistent with our findings. Evidence from Saudi Arabia study showed that of all study population with CO poisoning, 17% developed DNS [5]. Although this is higher than our findings (6%), this could be impacted by the low sample size of the earlier study (4 out of 24 patients developed DNS) [5].

### Evidence from international literature

Findings from broader literature (Asian and European studies) showed that the proportion of males with CO poisoning, compared to females, was lower or almost equal (ranging between 45 to 49%), inconsistent with our findings (64.7% were male) [5, 8, 16–18]. This might be explained by the latest report from the General Authority of Statistics in Saudi Arabia showing that 62.2% of people living in Saudi Arabia are males, compared to 38.8% females according to the 2023 Saudi Census [19].The mean age of patients presenting with CO poisoning differed between countries ranging from 29 (±17) to 44.56 (±16.08) years [8, 16–18].The lower mean age of patients with CO poisoning in Saudi Arabia might be attributed to the fact that mean age of people living in Saudi Arabia is 29 years, according to the 2023 Saudi Census [19].

Winter season was reported to be the most common season for CO poisoning cases [16, 17].

Furthermore, most CO poisoning cases occurred at home [17, 18], commonly due to stove (93%), gas heaters/cookers (5%) [18]. These findings were consistent with our findings showing most cases occurred in winter season and in enclosed spaces [18].

International evidence reported CNS manifestation to be the most common signs/symptoms associated with CO poisoning, similar to our findings [8, 17]. Headache was the most common reported manifestation. Other manifestations included nausea, dizziness, transient loss of consciousness, syncope, chest pain, and dyspnea.

Of CO poisoning patients, 11.3% developed DNS as reported by a study conducted in China, showing similar findings with our study reporting low proportions of patients developing DNS post CO exposure (6%) [18]. However, a study from Italy showed higher proportions (24.1%) of patients who developed DNS [8]. There is still no clear reason explaining the difference in the proportion of patients who developed DNS following CO exposure, but setting may contribute to this as differences between various study settings were reported in this study.

Sex was not shown to be associated with developing DNS [8, 18]. However, our study showed that women were higher than men to develop DNS. This might be impacted by lower sample size in our study and, therefore, larger sample size is needed to assess the association of sex with developing DNS post CO poisoning.

For the development of clinical manifestations, patients who developed DNS commonly had transient loss of consciousness (65%) and headache (56%), consistent with our findings [8].

### Strengths, limitations, and recommendations

This study is one of the few studies nationally characterizing patients presenting at the ED with CO poisoning and the association of CO poisoning with developing DNS. The findings of this study could add great value to international literature especially in the middle east region. Data of this study was collected from one of the largest EDs of tertiary hospitals in the middle east. All patients presenting at the ED with CO poisoning were included for better description of their characteristics at ED attendance. However, the study has some evident limitations that need to be highlighted. First, the retrospective nature could affect the study findings as we were unable to collect data of some important variables such as weather condition, smoking status, and Glasgow Coma Scale (GCS) score. Furthermore, although data was collected over 6-year period, the sample size of the population was relatively low (85 patients) especially for patients who developed DNS (6% of the study population), potentially impacting the significance of our study findings. Moreover, data was collected from a single center which could affect the applicability of the study findings to the Saudi population.

The findings of this study highlighted the need to conduct a prospective large-scale multicenter study to identify predictive risk factors for the development of DNS. The current findings did not show any risk factor that is significantly associated with DNS except sex. The study findings also highlight the need to raise public awareness about the causes of in-home CO poisoning especially during winter period as most CO poisoning cases were reported in this period of the year.

## Conclusion

This study has described the demographics and clinical characteristics of patients with CO poisoning. Seventy-two percent of the patients were adults and 65% were males. Clinical manifestations related to the central nervous system. Gender, BMI, and age group were significantly associated with COHb level. Few patients (6%) developed DNS following CO intoxication. Most common signs and symptoms were related to the central nervous system in 40% of the cases. Eighty percent of the cases developed DNS in the period 2 to 10 days after CO intoxication. Our study has not found any statistically significant association between demographic and clinical characteristics with the development of DNS. This is potentially due to the lower proportions of patients who developed DNS following CO intoxication. Further larger-scale multicenter studies are needed to assess the factors associated with the development of DNS for patients with CO poisoning.

## Data Availability

The datasets used and/or analysed during the current study are available from the corresponding author on reasonable request.

## Abbreviations

CO: Carbon Monoxide
USA: United States of America
COHb: Carboxyhemoglobin level
WHO: World Health Organization
GCS: Glasgow Coma Scale
DNS: Delayed Neuropsychiatric Sequelae
ED: Emergency Department
KAMC: King Abdulaziz Medical City
KASCH: King Abdullah Specialist Children’s Hospital
BMI: Body Mass Index
CNS: Central Nervous System
ABG: Arterial Blood Gas
KAIMRC: King Abdullah International Medical Research Center
IQR: Inter-quartile Range
